# Revisiting the Design of Phase III Clinical Trials of Antimalarial Drugs for Uncomplicated Plasmodium falciparum Malaria

**DOI:** 10.1371/journal.pmed.0050227

**Published:** 2008-11-18

**Authors:** Steffen Borrmann, Tim Peto, Robert W Snow, Win Gutteridge, Nicholas J White

## Abstract

Steffen Borrmann and colleagues discuss appropriate endpoints and their measurement during phase III trials of new antimalarial drugs.

In 2002, Plasmodium falciparum caused at least 0.5 billion uncomplicated clinical attacks of malaria worldwide, particularly in non-immune young children living in Africa [1]. Because inadequately treated uncomplicated P. falciparum malaria can progress rapidly to life-threatening severe malaria [2,3], mortality from P. falciparum in Africa doubled during the 1990s against a rising frequency of resistance to commonly used drugs, such as chloroquine and sulfadoxine-pyrimethamine [4].

In recent years, the deployment of highly efficacious oral three-day regimens of artemisinin-based combination therapies (ACTs) with parasitological cure rates of 95% or greater in more than 40 endemic countries has radically changed antimalarial treatment [5–8]. This success demands a new approach to the ways in which we assess new antimalarial drugs during clinical development and judge their potential utility for the public health deployment [9]. For example, the ability of slowly eliminated new drugs to delay re-infections and thus secondary malaria episodes for several weeks by suppressing the growth of P. falciparum provides additional public health benefits, especially in high-transmission areas where re-infection rates can exceed 50% in less than six weeks [10,11]. In turn, new drugs with comparatively higher efficacy against primary blood stage infections and/or a shorter elimination half-life minimise morbidity from recrudescent primary infections and may reduce the rate of spread of resistance [9,12], and are thus particularly valuable in situations of low or decreasing transmission rates [13] where the likelihood of re-infection during the relatively short post-treatment prophylactic period is lower (Figure 1A and 1B). There is already a general consensus on the design and interpretation of clinical trials used for monitoring antimalarial drug efficacy by national malaria control programmes [14]. The objective of this paper is to reflect on the design and interpretation of phase III trials.

**Figure 1:**
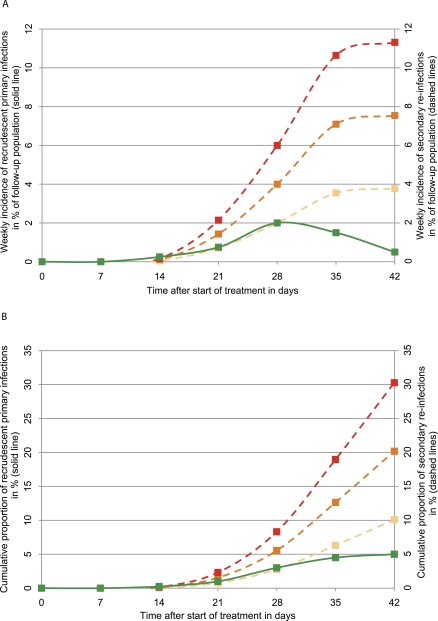
Simulated Plots of Weekly Incidence Rates and Cumulative Proportions for Recrudescent Primary Infections and Re-Infections Following Treatment of Uncomplicated P. falciparum Malaria with a Slowly Eliminated (Partner) Drug (Half-Life of ~1 Week) The solid line represents recrudescent infections with a cumulative failure rate of 5% by day 42. Dashed lines represent different rates of re-infections corresponding to entomological inoculation rates of two (yellow), four (orange), and six (red) infective mosquito bites/year, respectively. Trailing plasma drug concentrations delay both detectable recrudescent primary and secondary blood stage infections—however, both effects are fading until day 42. Figure 1A illustrates that most recrudescent primary infections are captured by day 42. Extension of follow-up beyond day 28 results in increased ratios of new versus recrudescent infections and hence, elevated risk of outcome misclassifications due to intrinsic limitations of current molecular techniques used to discriminate between primary and secondary infections. The assessment of the total number of recurrent infections as a composite outcome (often denoted the PCR “uncorrected cure rate”) requires some time limits as re-infection occurs eventually in almost everyone after blood concentrations of the drug(s) fall below the MIC (Figure 1B).

**Summary Points**

- Curing malaria episodes is the key priority for antimalarial treatment; its measurement as primary endpoint in phase III trials provides consistent estimates of the antiparasitic effect of a new regimen.
- The delay of secondary malaria episodes by slowly eliminated new drugs provides additional public health benefits in high-transmission areas; it should therefore be measured as key secondary or, preferably, co-primary endpoint.
- WHO suggests aiming to achieve parasitological cure rates of ≥95%; if adopted for drug development this sets a high bar and may lead to the premature rejection of potentially valuable new drugs.
- Since small differences in cure rates of ≥95% may be outweighed by advantages in cost, dosing, or tolerability, new drugs can be examined in non-inferiority trials with a proposed difference margin of ≤5%.
- We recommend survival analysis of the primary endpoint.

## Defining the Primary Endpoint

In the era of highly efficacious ACTs there is considerable debate among experts on what, exactly, constitutes the most relevant property of a new antimalarial drug [9,15]. In other words, in phase III trials, should we ask:

1. How efficacious is the new drug in curing primary blood stage infections (chemotherapeutic efficacy)?
  or
2. How efficacious is the new regimen in curing primary infections *and* in preventing secondary infections (composite chemotherapeutic and post-treatment prophylactic efficacy)?
  or
3. How efficacious is the new drug in reducing the post-treatment incidence of malaria and its complications (clinical risk reduction)?

Whilst the scientific question being asked determines the definition of the primary endpoint, it is important to understand that the measure of drug efficacy by any of these three endpoints is, to a variable extent, determined by factors unrelated to the intrinsic antiparasitic effects of antimalarial treatment regimens. Extensive inter-population variation in levels of acquired host immunity [16] and parasite re-infection rates [10,17] complicates the interpretation and comparison of results from different geographical areas or between sites in multicentre phase III trials [18]. Pharmacogenetic differences have not been shown to play a major role in the variation in therapeutic response (although there are relatively few studies), but pharmacokinetic differences related to age and pregnancy have been large, and clinically important for several antimalarials [19]. This is a concern for malaria control programmes in endemic countries and agencies that fund drug purchases, both of whom require comparability of clinical trial outcomes to weigh the relative advantages of new antimalarial drugs as they emerge from the clinical development pipeline (phases I–IV; Figure 2).

**Figure 2:**
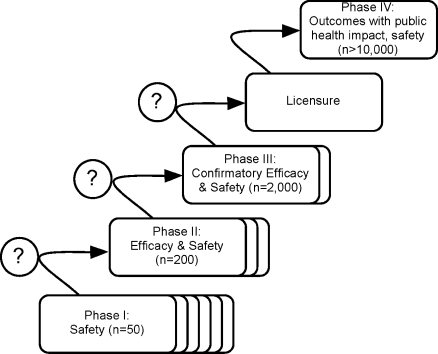
Simplified Outline of the Iterative Process of the Clinical Development of New Antimalarial Candidate Drugs or Drug Combinations Phase III trials are designed to provide pivotal efficacy and safety data for obtaining regulatory approval. The indicated numbers of study participants are approximations of the magnitude of required total sample sizes in different transmission settings.

### 1. Cure of primary malaria episodes and infections.

The chemotherapeutic efficacy of an antimalarial drug against primary malaria episodes can be estimated by the established “in vivo test” methodology [20]. This test observes two key events. The first criterion is the alleviation of clinical symptoms and the suppression of the density of the pathogenic asexual blood stage parasites in the peripheral blood below the light-microscopic detection threshold (around 20–50 parasites/μl) within the first few days (avoiding “early treatment failure”). The second event is the potential recrudescence of persistent asexual blood stage parasites after one week (“late parasitological treatment failure”) (Figure 1A), which may or may not be associated with clinical symptoms of malaria (“late clinical treatment failure”) [18,20]. In the absence of re-infection, and assuming adequate drug absorption, the incidence of and the time to the microscopic detection of recrudescent blood stage infections has been shown to be primarily a function of (1) variability in pharmacodynamic variables, i.e., parasite susceptibility and initial parasite biomass; (2) pharmacokinetic parameters, i.e., drug elimination kinetics; and also (3) drug-unrelated parameters, primarily the patients' immune status [16,20–23].

As many drugs have no effects on pre-erythrocytic (liver stage) development, new blood stage infections may become patent as early as one week after blood concentrations of the antimalarial drug fall below the minimum inhibitory concentration (MIC) [24]. This situation requires molecular fingerprinting techniques to determine the likely origin of the parasite strain(s) in the recurrent infection and hence, to separate chemotherapeutic failures (recrudescence of the primary infection) from reinfection (Figure 1A) [14]. Since even a single misclassification can significantly change the risk difference between test and reference arms at cure rate estimates of at least 95%, the issue of accuracy of parasite strain genotyping (“PCR correction”) has attracted considerable attention [9,25–29]. The controversy centres on the very real risk that a variable proportion of recurrent infections may either (1) be misclassified leading to underestimation or (less likely) overestimation of cure rates or (2) remain indeterminate (unclassifiable) [29,30], especially in high-transmission areas when follow-up periods extend past day 28 (Figure 1A) [25–28]. There are also unresolved issues related to relapses of P. vivax infections in Asia [31]. Even so, re-infection-adjusted parasitological cure rates provide demonstrably consistent estimates of the antiparasitic effect of an antimalarial regimen across different transmission settings [10,17], and the World Health Organization (WHO) endorses PCR-corrected primary endpoints for monitoring antimalarial drug efficacy in endemic countries [14,32]. At the same time, the exclusion or censoring of all reinfections limits the clinical relevance of chemotherapeutic endpoints in high-transmission areas (Figure 1B).

### 2. Cure of primary infections and prevention of re-infections.

Of course, any recurrent infection can be considered a failure, as even new infections reflect a post-treatment “chemoprophylaxis” breakthrough (Figure 1A) [33]. Frequently, this effect has been assessed by using the proportion of the total number of recurrences as a composite endpoint without distinguishing between recrudescent primary and secondary infections (or for that matter, curative and preventive effects) [15,34]. But since re-infection occurs eventually in almost everyone after blood concentrations of the drug(s) fall below the MIC, the measurement of the composite endpoint requires some time limits.

In high-transmission areas, the composite effect size will largely be determined by the post-treatment prophylactic efficacy against secondary infections [10]. The duration of this protection depends on the ability of the drug to suppress the clinically silent intra-hepatocytic parasite development and on the elimination half-life of the drug (Figure 1A) [33]. The problem with assessing the composite outcome (often denoted the PCR “uncorrected cure rate”) alone is that it combines two effects (curative activity and post-treatment prophylaxis) that, although related, are not directly proportional to each other (Figure 1B). To illustrate this point, the reported crude, PCR-uncorrected parasitological day 28 failure rates in two trials of the recently registered six-dose regimen of artemether-lumefantrine can be compared. These rates differed by 25% whereas the corresponding parasitological cure rate estimates adjusted for the difference in re-infection rates varied by only 7% [10,17]. The interpretation of such large inter-site variations poses a problem in rigorous and costly phase III programs where the reproducibility of what the phase III trial set out to measure, i.e., the antiparasitic effect of the new regimen, is a prime concern [14].

### 3. Post-treatment reduction of clinical risk.

Recrudescent primary infections, as well as re-infections, entail the risk of secondary malaria and/or hematological complications requiring re-treatment [34,35]. Clinical endpoints, which measure the reduction of these clinical events, provide important public health–relevant information above parasitologically defined endpoints [15,36,37].

The risk that a patient with recurrent infection will be symptomatic at detection or succumb subsequently to secondary malaria during follow-up is related to the individual level of specific, if imperfect, acquired immunity (“semi-immunity”). For example, an analysis of antimalarial treatment trials in high-transmission areas found that children below one year were at least three times as likely to experience symptomatic recurrences than children aged more than three years [34]. This dependence of clinical risks on the prevalence of acquired immunity in the study population or age group [18,34] (on top of large variation introduced by different reinfection/recurrence rates) confounds the estimation of the antiparasitic efficacy of an antimalarial drug by clinical endpoints, and therefore undermines the comparability of study results between different endemic areas, leading to large inter-site variation in multicentre phase III trials [18]. Alternatively, preliminary data from study sites on key confounding factors could, in conjunction with normalisation provided by a standard comparator treatment, be used to adjust for inter-site differences in trials with a primary clinical endpoint. The feasibility of such an adjusted primary analysis approach in regulatory phase III trials needs to be explored further.

Based on the above considerations, whilst it is clear that, in areas of very high transmission, multiplicity of infection confers a significant and irreducible error in genotyping [38], parasite “strain typing” with the highest possible resolution power is required for separating curative and preventive effects in regulatory phase III trials of new antimalarial drugs. Chemotherapeutic efficacy against primary malaria episodes and infections should be the primary endpoint of phase III trials; protective efficacy against secondary infections and clinical episodes should be either key secondary endpoints, or in high-transmission areas, possibly co-primary endpoints. A broad consultation on the utility, classification, and respective merits of alternative, especially PCR-uncorrected, composite endpoints in phase III trials of new antimalarial drugs, including measurement of the *delay* instead of proportions of recurrent infections, should be undertaken.

## Defining the Benchmark for Efficacy

Optimal target profiles for new antimalarial drugs have been described elsewhere and used as guiding principles from early discovery through clinical development [39,40]. In an ideal world, antimalarial treatments would be 100% efficacious; in the real world, WHO suggests aiming to achieve parasitological cure rate point estimates of at least 95% (excluding re-infections) [41]. To establish with 95% confidence that a new treatment can demonstrate a cure rate of at least 95% in a phase III trial with 500 patients per group, the true (unknown) cure rate would have to be at least 97%. This sets a high bar for evaluating new candidate drugs and might lead to the premature rejection of new products because of suboptimal dosing, formulation, or the play of chance. A greater than 90% parasitological cure rate (lower boundary of the 95% confidence interval) represents an alternative, more realistic initial target for new treatments (requiring only a true cure rate of at least 93% under the same sample size assumption).

To date, there is no guidance on the benchmark for the protective efficacy of a new antimalarial drug in preventing secondary infections, or the composite efficacy of curative and preventive effects. The establishment of a uniform benchmark is challenging: in addition to clearing more than 90% of primary infections, should a new drug prevent 30%, 40%, or more of secondary infections? Over which time frame—four, six, or more weeks? Or, put more simply, for how long on average should re-infections or, more generally, recurrent infection be *delayed* ? Should the new drug be superior to or “just as good as” the reference treatment? Or to use a hypothetical example, in times of continued shortage of viable therapeutic options, is it desirable to turn down a new once-daily regimen with a composite efficacy comparable to the recently introduced twice-daily regimen of artemetherlumefantrine, but which may be inferior to dihydroartemisinin-piperaquine because piperaquine is exceptionally slowly eliminated [10,42,43]? All this needs to be considered in a context of declining malaria transmission as effective control measures are rolled out. There is currently no consensus on these issues.

## Duration of Follow-Up

Figure 1A illustrates how trailing plasma drug concentrations of slowly eliminated antimalarial drugs delay detectable recrudescent primary and secondary blood stage infections alike. These effects fade during follow-up beyond day 28.

The current use of day 28 estimates provides a single benchmark period to compare all new drugs, but we feel there is a need to revisit this single time-point for phase III trial endpoints, particularly for new investigational drugs or combinations containing at least one component with an intrinsically long plasma half-life.

## Superiority or Non-Inferiority?

In phase III, the new candidate drug is compared in a randomised controlled trial [44] to a standard treatment that retains the desired “control effect size” [44], e.g., the targeted more than 90% parasitological cure rate (excluding reinfections). Historically, when existing recommended treatments were failing, antimalarial phase III drug trials have been designed as superiority trials [9,45], but there is a dramatic increase in the sample sizes required to demonstrate superiority of a potential future replacement for a drug with currently very high parasitological cure rates [9].

Small differences in parasitological cure rates demonstrated in superiority trials, especially between 95% and 100%, are important in reducing the emergence and spread of resistance [9,12], but from a pragmatic perspective such differences may be outweighed by advantages in cost, dosing, shelf lives, or tolerability, or importantly, by the gain of a therapeutic alternative should parasite resistance to the current first-line drug emerge. An alternative to the superiority criterion for a new drug is whether it is “no worse than” the standard treatment [9]. Non-inferiority trials test if the observed difference between treatment arms falls below a difference Δ margin; if not, the new treatment is considered non-inferior [46]. The Δ margin is selected to ensure that a drug with clinically meaningful inferiority to the comparator treatment is rejected [47]. We propose to use a Δ margin of 5%, or its equivalent as hazard ratio limit, within the limits of cure rates exceeding 90%.

The *superiority* of the post-treatment prophylactic efficacy of a new drug with comparatively higher anti-liver stage activity or prolonged plasma half-life can be best examined in areas with high re-infection rates because of the postulated differential impact on re-infection and/or secondary malaria rates [33]. The comparison to a reference treatment with similar pharmacodynamic and pharmacokinetic characteristics, however, requires inflated sample sizes in high-transmission areas.

**Linked Perspective**

This Policy Forum is further discussed in a *PLoS Medicine* Perspective by Colin J. Sutherland:

Sutherland CJ (2008) Comparing highly efficacious antimalarial drugs. PLoS Med 5(11): e228. doi:10.1371/journal.pmed.0050228

## Analytical Strategies

### Intention-to-treat versus per-protocol approach.

The transition from superiority to non-inferiority trial designs has implications for the choice of the primary analysis population [48]. Non-inferiority trials lack internal controls for assessment accuracy, a conservative analysis approach, and protection from bias by blinding [9]. Technical and methodological inadequacies, e.g., missing or uninterpretable PCR data in an intention-to-treat analysis (considered as conservative in superiority trials), or simple mistakes can blur true differences in treatment effects and thus lead to inadvertent conclusion of *non-inferiority* when in fact the new drug is inferior (type I error) [46,49]. On the other hand, indiscriminate classification of defaulting patients as failures in the intention-to-treat analysis (“worst-case scenario”), particularly losses to follow-up or re-infections when recrudescent rates or time to recrudescence are the primary endpoint, reduces the power of non-inferiority trials and thus leads to incorrect finding of *inferiority* when in fact the new drug is *not* inferior (type II error). If the primary endpoint is measured as proportional point estimate (i.e., not by survival analysis), we recommend basing the analysis on the per-protocol population, which includes only patients with observed treatment responses and other informative outcomes, e.g., intake of outside-protocol antimalarial medication. The preferred method for comparing antimalarial drug efficacy, however, is survival analysis [9].

### Survival analysis.

Survival analysis techniques are increasingly used to assess failure rates in randomised phase III trials [49]. The key advantages of this approach for antimalarial, or any other anti-infective drug studies, are: (1) the statistical model reflects specific biological processes (delay of recrudescence or re-infection [20]) and (2) the analytical procedure deals specifically with incomplete but informative data, i.e., patients with incomplete follow-up contribute to the assessment, whereas they are excluded in per-protocol analyses [9,50]. Reinfections, losses to follow-up, and protocol deviations can be censored at the time of defaulting. Analogous to tests of the risk difference of point estimates [37], the non-inferiority hypothesis of survival estimates can be examined by using a modified confidence interval (CI) approach [51].

## Interpretation of Trial Results

The interpretation of a non-inferiority trial with a survival estimate of recrudescent infections can be based on two criteria: (1) the hypothesis test result, e.g., the 95% CI of the proportional hazard ratio test/control remains below the non-inferiority limit on the hazard ratio to support claims of efficacy [51]; and (2) the day 28 and later survival rate estimate in the test drug arm. Table 1 lists possible combinations of trial results and recommendations for their interpretation. The standardised reporting of key baseline and outcome variables of antimalarial trials using a hierarchical system will greatly facilitate further detailed post-hoc analyses and interpretations [18].

**Table 1:**
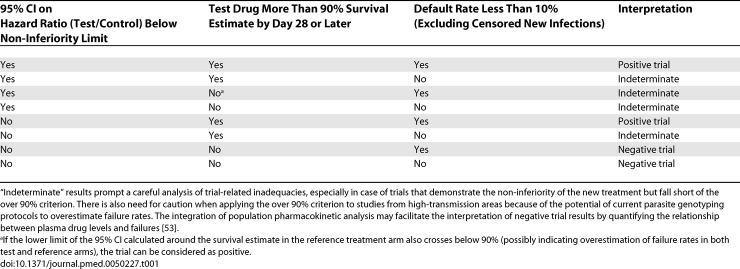
Potential Outcome Scenarios of Antimalarial Phase III Trials with a Chemotherapeutic Endpoint and Their Proposed Interpretation

## Conclusion

Consensus-agreed regulatory guidelines on how phase III trials of antimalarial drugs for uncomplicated P. falciparum malaria are designed and interpreted are only now being developed [52]. This review intends to stimulate an informed discussion on the utility of the different primary endpoints in future phase III antimalarial trials and proposes a comparative framework for the interpretation of results from ongoing trials. We hope that consensus between academia, public health professionals, industry, and regulators on the design and particularly the primary endpoint of phase III trials allows earlier appraisal of the potential advantages or equivalence of a new treatment in comparison with existing therapies ahead of more extensive phase IV programmes.

## Abbreviations

ACT: artemisinin-based combination therapy
CI: confidence interval
MIC: minimum inhibitory concentration
WHO: World Health Organization
